# Empagliflozin decreases myocardial cytoplasmic Na^+^ through inhibition of the cardiac Na^+^/H^+^ exchanger in rats and rabbits

**DOI:** 10.1007/s00125-016-4134-x

**Published:** 2016-10-17

**Authors:** Antonius Baartscheer, Cees A. Schumacher, Rob C. I. Wüst, Jan W. T. Fiolet, Ger J. M. Stienen, Ruben Coronel, Coert J. Zuurbier

**Affiliations:** 1 Department of Clinical and Experimental Cardiology, Academic Medical Center, University of Amsterdam, Amsterdam, the Netherlands; 2 Department of Physiology, Institute for Cardiovascular Research, VU University Medical Centre, Amsterdam, the Netherlands; 3 Laboratory Genetic Metabolic Diseases, Academic Medical Center, University of Amsterdam, Amsterdam, the Netherlands; 4 Department of Physics and Astronomy, Faculty of Science, VU University, Amsterdam, the Netherlands; 5 University of Bordeaux, L’Institut du Rythmologie et Modélisation Cardiaque (LIRYC), Bordeaux, France; 6 Department of Anesthesiology, Laboratory of Experimental Intensive Care and Anesthesiology, Academic Medical Center, University of Amsterdam, Meibergdreef 9, 1105 AZ Amsterdam, the Netherlands

**Keywords:** Calcium, Cardiac death, Diabetes, Glucose, Heart failure, Sodium

## Abstract

**Aims/hypothesis:**

Empagliflozin (EMPA), an inhibitor of the renal sodium–glucose cotransporter (SGLT) 2, reduces the risk of cardiovascular death in patients with type 2 diabetes. The underlying mechanism of this effect is unknown. Elevated cardiac cytoplasmic Na^+^ ([Na^+^]_c_) and Ca^2+^ ([Ca^2+^]_c_) concentrations and decreased mitochondrial Ca^2+^ concentration ([Ca^2+^]_m_) are drivers of heart failure and cardiac death. We therefore hypothesised that EMPA would directly modify [Na^+^]_c_, [Ca^2+^]_c_ and [Ca^2+^]_m_ in cardiomyocytes.

**Methods:**

[Na^+^]_c,_ [Ca^2+^]_c_, [Ca ^2+^]_m_ and Na^+^/H^+^ exchanger (NHE) activity were measured fluorometrically in isolated ventricular myocytes from rabbits and rats.

**Results:**

An increase in extracellular glucose, from 5.5 mmol/l to 11 mmol/l, resulted in increased [Na^+^]_c_ and [Ca^2+^]_c_ levels. EMPA treatment directly inhibited NHE flux, caused a reduction in [Na^+^]_c_ and [Ca^2+^]_c_ and increased [Ca^2+^]_m_. After pretreatment with the NHE inhibitor, Cariporide, these effects of EMPA were strongly reduced. EMPA also affected [Na^+^]_c_ and NHE flux in the absence of extracellular glucose.

**Conclusions/interpretation:**

The glucose lowering kidney-targeted agent, EMPA, demonstrates direct cardiac effects by lowering myocardial [Na^+^]_c_ and [Ca^2+^]_c_ and enhancing [Ca^2+^]_m_, through impairment of myocardial NHE flux, independent of SGLT2 activity.

**Electronic supplementary material:**

The online version of this article (doi:10.1007/s00125-016-4134-x) contains peer-reviewed but unedited supplementary material, which is available to authorised users.

## Introduction

The recent Empagliflozin, Cardiovascular Outcomes, and Mortality in Type 2 Diabetes (EMPA-REG OUTCOME) study has demonstrated that empagliflozin (EMPA), an inhibitor of renal sodium-glucose cotransporter (SGLT)2, resulted in a 38% reduction in the relative risk of cardiovascular death and a 35% risk reduction of hospitalisation for heart failure in patients with type 2 diabetes [1]. SGLT2 inhibitors also result in increased urinary glucose excretion in diabetic patients. The mechanisms behind these effects are unknown [1, 2], however, since these findings do not suggest any beneficial effects of EMPA on the incidence of myocardial infarction and stroke, it is unlikely that they can be ascribed to a general reduction in risk factors for cardiovascular disease (e.g. glycaemic status, body weight, blood pressure). We hypothesised that EMPA has a direct cardiac effect; since increases in myocardial intracellular Na^+^ and Ca^2+^ concentrations are early hallmarks and drivers of cardiovascular death and heart failure [2–6], we investigated whether EMPA (1) directly reduces intracellular cardiac cytoplasmic Na^+^ ([Na^+^]_c_) and Ca^2+^ ([Ca^2+^]_c_) concentration; (2) affect the (upstream) cardiac Na^+^/H^+^ exchanger (NHE); and (3) changes (downstream) mitochondrial Ca^2+^ concentration ([Ca^2+^]_m_).

## Methods

Animal handling was in accordance with the Institutional Animal Care and Use Committee of the VU Medical Center and Academic Medical Center Amsterdam, and was conducted according to the *Guide for the Use and Care of Laboratory Animals*.

For detailed methods, see electronic supplementary material [ESM] Methods. Cardiomyocytes were isolated from hearts from healthy rabbits and rats. Cells were left untreated or treated with 1 μmol/l EMPA (MedChem Express, Monmouth Junction, NJ, USA), 5.5 mmol/l or 11 mmol/l glucose (Sigma-Aldrich Chemie, Zwijndrecht, the Netherlands), 10 μmol/l cariporide (Aventis Pharma, Frankfurt, Germany) or 20 mmol NH_4_Cl (NH^+^) (Sigma-Aldrich Chemie), either alone or in combination. [Na^+^]_c_ and [Ca^2+^]_c_ were fluorometrically measured in rabbit cardiomyocytes using SBF1 and indo-1, respectively, at 2 Hz field stimulation. NHE activity was measured in rabbit cardiomyocytes by recording SNARF fluorescence following an NH_4_^+^ pulse. Using adenoviral transfection, a ratiometric mitochondrially targeted fluorescence resonance energy transfer (FRET)-based Ca^2+^ indicator (4mtD3cpv, MitoCam) was expressed in rat cardiomyocytes and free [Ca^2+^]_m_ was measured using the fluorescence ratio, yellow fluorescent protein intensity/cyan fluorescent protein intensity (YFP/CFP), in cultured adult rat cardiomyocytes.

### Statistics

Data are reported as mean ± SE. Kolmogorov–Smirnov normality testing was applied to decide the use of non-parametric vs parametric testing. Mann–Whitney tests were used to evaluate the effects of glucose on Na^+^ and Ca^2+^. ANOVA with Dunnett’s post hoc tests was used to compare group means with the control ([EMPA] 0) group. Additionally, unpaired *t* tests were used to evaluate EMPA effects on [Ca^2+^]_c_ and EMPA effects on the NHE flux in the absence of glucose, ANOVA with post hoc testing with Bonferroni corrections were used to compare cariporide and EMPA effects on the NHE flux in the presence of glucose and two-way ANOVA for repeated measures followed by post hoc contrast with Bonferroni correction at one time point were performed to detect EMPA effects on [Ca^2+^]_m_.

## Results

### EMPA decreases [Ca^2+^]_c_ and [Na^+^]_c_ and increases [Ca^2+^]_m_

**Fig. 1:**
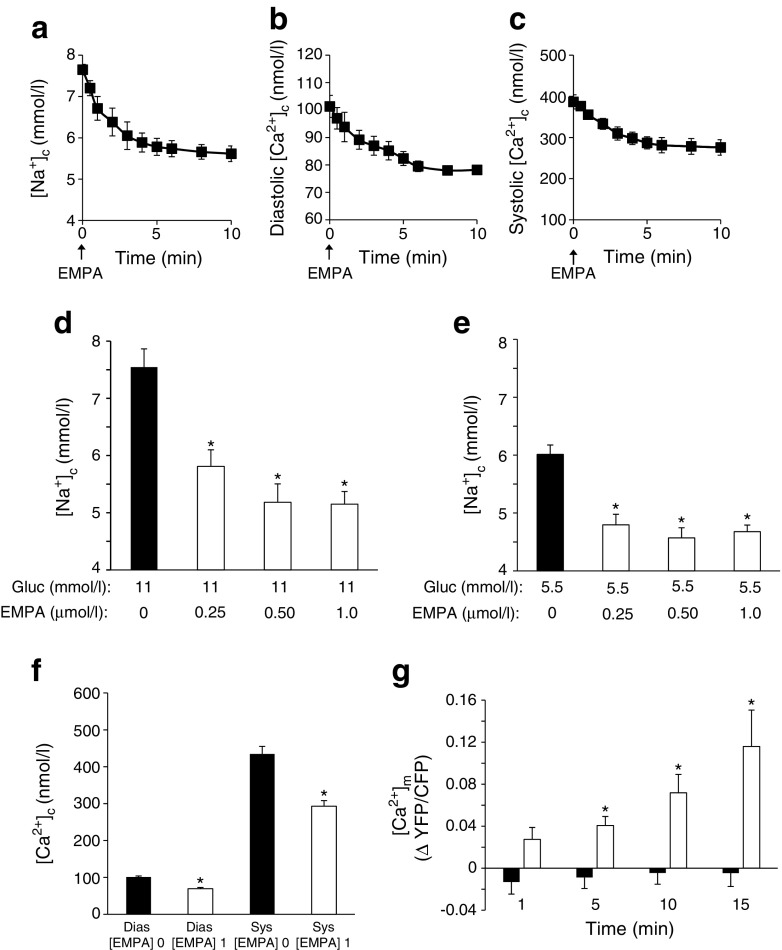
EMPA effects on [Na^+^]_c_, [Ca^2+^]_c_ and [Ca^2+^]_m_. (**a–c**) EMPA (1 μmol/l) acutely lowers (**a**) [Na^+^]_c_, and (**b**) diastolic [Ca^2+^]_c_ and (**c**) systolic [Ca^2+^]_c_ in rabbit cardiomyocytes. (**d**,**e**) Effects of EMPA (3 h incubation) on [Na^+^]_c_ at (**d**) 11 mmol/l and (**e**) 5.5 mmol/l glucose (Gluc), respectively. *p < 0.05 vs 0 μmol/l EMPA, ANOVA with Dunnett’s post hoc tests. (**f**) Effects of EMPA (3 h pre-incubation; [EMPA] 1) on diastolic (Dias) and systolic (Sys) [Ca^2+^]_c_ at 11 mmol/l glucose. *p < 0.05 vs 0 μmol/l EMPA ([EMPA] 0), unpaired *t* test. (**g**) [Ca^2+^]_m_ as determined by the change in fluorescence ratio, YFP/CFP, relative to 0 min (*t* = 0) during a 15 min incubation with 1 μmol/l EMPA (white bars) or vehicle (black bars). *p < 0.05 vs vehicle at a similar time point, two-way ANOVA for repeated measures followed by post hoc contrast with Bonferroni correction at one time point. For (**a**–**c**) *n* = 6–8 cells from 3 rabbits per investigation; for (**d**–**f**), *n* = 20–30 cells from 3 rabbits per investigation; for (**g**) *n* = 8–9 cardiomyocytes from 2–3 rats for each group (EMPA or vehicle)

First, we examined the acute effects of EMPA in isolated rabbit cardiomyocytes in the presence of 11 mmol/l glucose. EMPA (1 μmol/l) decreased [Na^+^]_c_ within 10 min (Fig. 1a). Vehicle administration had no effect (data not shown). In addition, similar acute EMPA effects were observed for diastolic and systolic [Ca^2+^]_c_ (Fig. 1b,c).

Extended (3 h at 37°C in 3 ml HEPES buffer) EMPA incubation at clinically relevant concentrations (0.25–1 μmol/l) also reduced [Na^+^]_c_, in the presence of both 11 mmol/l (Fig. 1d) and 5.5 mmol/l (Fig. 1e) glucose. Increasing glucose concentration from 5.5 mmol/l to 11 mmol/l significantly increased [Na^+^]_c_ (6.0 ± 0.2 mmol/l to 7.6 ± 0.3 mmol/l; *p* < 0.001; Fig. 1d), diastolic [Ca^2+^]_c_ (68 ± 3 nmol/l to 101 ± 3 nmol/l; *p* < 0.001) and systolic [Ca^2+^]_c_ (331 ± 18 nmol/l to 435 ± 20 nmol/l; *p* < 0.001) (data not shown). EMPA preincubation of cardiomyocytes at 11 mmol/l glucose also significantly lowered diastolic and systolic [Ca^2+^]_c_ (Fig. 1f). Patch clamp experiments (*n* = 4 cells) demonstrated that 1 μmol/l EMPA had no effect on action potential duration (data not shown). The downstream effects of the cytosolic changes of [Na^+^]_c_ and [Ca^2+^]_c_ on [Ca^2+^]_m_ were subsequently tested. Acute EMPA (1 μmol/l) administration significantly increased [Ca^2+^]_m_ (Fig. 1g).

Collectively, these data show that EMPA reduces myocardial [Na^+^]_c_ and [Ca^2+^]_c_ and increases [Ca^2+^]_m_, whereas increases in glucose are associated with elevated [Na^+^]_c_ and [Ca^2+^]_c_.

### Empagliflozin impairs cardiac NHE activity without SGLT involvement

**Fig. 2:**
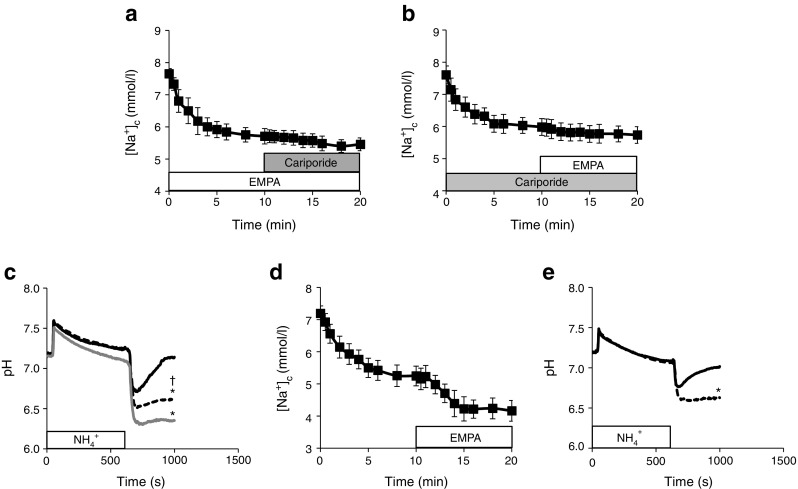
The effects of EMPA on the NHE in the presence and absence of glucose. (**a**) Cariporide exerted little effect on [Na^+^]_c_ when preceded by EMPA inhibition. (**b**) Similarly, EMPA was of little effect on [Na^+^]_c_ when preceded by Cariporide. (**c**) pH traces for rabbit cardiomyocytes exposed to an acidic load (NH_4_^+^) and during recovery for control (black solid line), Cariporide-treated (grey line) or EMPA-treated (black dashed line) cells in the presence of 11 mmol/l glucose. *p < 0.05 vs control for pH measured at 1000 s; †*p* < 0.05 vs Cariporide for pH measured at 1000 s, ANOVA with post hoc testing with Bonferroni corrections. (**d**) EMPA (1 μmol/l) acutely lowers [Na^**+**^ **]**_**c**_ even in the absence of extracellular glucose (*n* = 6/3 rabbits). (**e**) EMPA effects on pH recovery in the absence of extracellular glucose (*n* = 6/3 rabbits). Control (*black solid line*), EMPA-treuted (*black dashed line*). *p < 0.05 vs control for pH measured at 1000 s, unpaired *t* test. *n* = 6 cells from 3 rabbits for all except for (**c**), where *n* = 5–6 cells from 4 rabbits

Because the rapid action of EMPA resembled that of the NHE-inhibitor Cariporide on [Na^+^]_c_ and [Ca^2+^]_c_ [3], we investigated whether EMPA has NHE-inhibiting properties. First, we examined EMPA and Cariporide interactions on [Na^+^]_c._ Following the reduction in [Na^+^]_c_ with EMPA, additional application of Cariporide had only a minimal effect on [Na^+^]_c_ (Fig. 2a). Similarly, following the reduction in [Na^+^]_c_ with 10 min pre-treatment with Cariporide, subsequent EMPA application had a minimal effect on [Na^+^]_c_ (Fig. 2b). Second, we examined NHE flux, as determined by measuring pH recovery after an acute acidic load via wash-out of NH_4_^+^. In control conditions, pH quickly recovered to normal values after NH_4_^+^ wash-out. However, this recovery of pH was totally inhibited by the specific NHE inhibitor Cariporide, reflecting reduced NHE activity (Fig. 2c). In the presence of EMPA, the NHE flux was also strongly reduced by approximately 80% of the reduction observed with Cariporide (Fig. 2c). During NH_4_^+^ application, no significant difference in pH recovery was observed between Cariporide and control (data not shown). Finally, we evaluated whether SGLTs were involved in the effects of EMPA on NHE by repeating the experiments in the absence of glucose; in glucose-free conditions, EMPA also reduced [Na^+^]_c_ (Fig. 2d) and impaired NHE flux (Fig. 2e).

These data suggest that EMPA affects intracellular ion homeostasis in the cardiomyocyte through direct interaction with the NHE, without the involvement of SGLTs.

## Discussion

The present study demonstrates for the first time that the kidney-targeted therapeutic agent, EMPA, directly reduces myocardial intracellular Na^2+^ through an interaction with the NHE, independent of cardiac SGLT inhibition. The observed decreased [Ca^2+^]_c_ and increased [Ca^2+^]_m_ are likely to have occurred secondary to the decrease in [Na^+^]_c_ via the sarcolemmal and mitochondrial Na^+^/Ca^2+^ exchanger (NCX), respectively [6, 7]. Dapagliflozin, another SGLT2 inhibitor with a slightly different chemical composition, was recently shown to reduce cell shortening after 5 min but not after 3 h of treatment. It was also found to reduce systolic but not diastolic Ca^2+^ in cardiomyocytes from models of type 1 diabetes (but not in control cardiomyocytes) [8]. In comparison with dapagliflozin, the current study indicates a stronger and longer lasting effect of EMPA on cardiomyocytes. It is suggested that further research is required to compare the effects of different classes of SGLT2 inhibitors on cardiomyocytes characteristics.

### EMPA: cardiac NHE and SGLT2

Our observations that the effects of EMPA were independent of glucose presence is in agreement with a previous observation that SGLT2 is absent from cardiac tissue [9]. However, SGLT1 is present in the heart [9, 10], potentially explaining the increase in [Na^+^]_c_ with increased circulating glucose. It was recently reported that SGLT1 within the diseased human heart is mainly localised in capillaries and not in cardiomyocytes [11]. This is in contrast with other observations that SGLT1 is localised in the T tubules of isolated cardiomyocytes [10]. In the current study, the effects of glucose on [Na^+^]_c_ were detected in isolated cardiomyocytes; therefore, it is unlikely that glucose release from the capillaries plays a role in this effect. However, further research is needed to study the role of SGLT1 within the intact heart. We used EMPA concentrations in the range of clinically measured plasma concentrations (≤1 μmol/l) [1, 12], well below the IC_50_ of SGLT1 (8.3 μmol/l) for EMPA [13]. Therefore, EMPA does not exert its cardiac effects through SGLT1inhibition.

### EMPA raises cardiac [Ca^2+^]_m_

Previous studies have demonstrated that increasing [Na^+^]_c_ results in decreased [Ca^2+^]_m_ through increased mitochondrial efflux via the mitochondrial NCX [7]. This is very likely to be the mechanism underlying the EMPA-induced elevation in [Ca^2+^]_m_, observed in the present study. Mitochondrial Ca^2+^ is considered to be an important activator of ATP synthesis and of the antioxidant enzymatic network [5, 14]. Knowing that in vivo mitochondrial impairment, energy deficiency and increased oxidative stress [5, 14, 15] are hallmarks of failing hearts, restoring [Ca^2+^]_m_ (e.g. increasing [Ca^2+^]_m_ that is otherwise reduced due to Na^+^ loading) is predicted to be beneficial in this condition. Indeed, in a recent study, it was demonstrated that increasing mitochondrial Ca^2+^ during heart failure development was associated with the prevention of sudden death and overt heart failure [5]. This is in contrast to conditions where [Ca^2+^]_m_ is already elevated, such as during reperfusion injury phenomena, in which further increases in [Ca^2+^]_m_ can be detrimental.

### EMPA and heart failure

The propensities for arrhythmias, oxidative stress and heart failure are all associated with, and at least partly driven by, intracellular cardiomyocyte Na^+^ and Ca^2+^ loading [2, 3, 5, 6, 14]. Hyperglycaemia (as reported in this study) and diabetes [10] also result in intracellular Na^+^ and Ca^2+^ loading, possibly contributing to the reported interaction between hyperglycaemia/diabetes and cardiovascular diseases.

Previous studies have demonstrated that chronic inhibition of NHE prevents or mitigates heart failure in animal models [16, 17]. Although clinical studies of NHE inhibition have been performed in the setting of acute coronary syndromes (and largely show a neutral effect) no clinical studies have been performed using NHE inhibition in the chronic setting of heart failure and diabetes. Therefore, these types of studies are awaited. We surmise that the beneficial cardiovascular effects of EMPA are, at least in part, attributed to NHE inhibition. However, the current results do not allow for the generation of conclusive statements about a positive or negative role on cardiac mitochondrial function following EMPA treatment. The next area that needs to be evaluated is whether EMPA does indeed result in beneficial functional cardiac effects, such as increased ATP production, oxygen consumption and/or antioxidant capacity. Whether the reductions in both cardiac [Na^+^]_c_ and [Ca^2+^]_c_ with EMPA treatment, and the downstream effect of elevated [Ca^2+^]_m_ contribute to the primary mechanisms underlying the cardiovascular benefits observed in the EMPA-REG OUTCOME trial needs to be examined in future research.

In conclusion, our data demonstrate that the kidney-targeted therapeutic agent EMPA has direct cardiac effects, decreases cardiac [Na^+^]_c_ and [Ca^2+^]_c_ and increases cardiac [Ca^2+^]_m_ via inhibition of the cardiac NHE.

## Electronic supplementary material

Below is the link to the electronic supplementary material.

ESM 1 (PDF 511 kb)

## Abbreviations

[Ca^2+^]_c_: Cytoplasmic Ca^2+^ concentration
[Ca^2+^]_m_: Mitochondrial Ca^2+^ concentration
EMPA: Empagliflozin
EMPA-REG OUTCOME: Empagliflozin, Cardiovascular Outcomes, and Mortality in Type 2 Diabetes
[Na^+^]_c_: Cytoplasmic Na^+^ concentration
NCX: Na^+^/Ca^2+^ exchanger
NHE: Na^+^/H^+^ exchanger
SGLT: Sodium-glucose cotransporter
YFP/CFP: Yellow fluorescent protein intensity/cyan fluorescent protein intensity
