# Supplementary material for: Empagliflozin decreases myocardial cytoplasmic Na+ through inhibition of the cardiac Na+/H+ exchanger in rats and rabbits
Source: Diabetologia. 2016 Oct 17;60(3):568–73. doi: 10.1007/s00125-016-4134-x (PMC6518059; doi:10.1007/s00125-016-4134-x)
Supplement: Supplementary file 1 — (PDF 511 kb) [file 125_2016_4134_MOESM1_ESM.pdf]

Supplemental materials

Fluorescence measurements in cardiomyocytes

Cardiomyocytes were isolated from hearts of male New Zealand White (Crl:KBL (NZW) rabbits (n=13; 11-12 week old, Charles River, Lyon, France) and male Wistar rats (n=4; 300 g; Charles River, Leiden, The Netherlands) as reported previously (1, 2). Animals were housed for at least one week, with a 12 h day/night cycle, and food and drinking water ad libitum.

Each measurement was performed for at least 5 times in separate cells, with cells per measurement at least derived from 3 different animals. Isolated cells were excluded when in a contracture state; only quiescent cells were used.

$[Na^+]_c$ and $[Ca^{2+}]_c$ were fluorometrically measured with SBF1 (Abcam, Cambridge, United Kingdom) and indo-1 (Abcam, Cambridge, United Kingdom), respectively, at 2 Hz field stimulation as reported previously (1-4). Before each individual experiment, cells were incubated at 37°C with 10 µmol/L Indo-1/AM or SBFI-AM for 30 and 120 min, respectively. Myocytes were washed twice with fresh HEPES solution (Ca^{2+}=1.3 mmol/L without albumin), and kept for another 15 minutes to ensure complete de-esterification.

Loaded myocytes were attached to a poly-D-lysine (0.1 g/l) treated cover slip placed on a temperature controlled (37°C) microscope stage of an inverted fluorescence microscope (Nikon Diaphot, Tokyo, Japan) with quartz optics. A temperature controlled perfusion chamber (height 0.4mm, diameter 10 mm, volume 30 µL), with two needles at opposite sides for perfusion purposes, was tightly positioned over the cover slip. The content of the chamber could be replaced within 100 ms. A quiescent single myocyte was selected and the measuring area was adjusted to the rod shaped surface of the myocyte with a rectangular diaphragm. Bipolar square pulses for field stimulation (30 V/cm) were applied at a frequency of 2 Hz through two thin parallel platinum electrodes at a distance of 8 mm. Dual wavelength emission fluorescence was recorded with and corrected for fluorescence of unloaded myocytes as described previously (3, 4).

The emission wavelengths for Indo-1 and SBFI were 410/510 and 410/590, respectively. Excitation wavelengths was 340 nm for both. Indo-1 fluorescence was recorded at 1 kHz during 500 ms. For each myocyte 5 calcium transients were averaged. SBFI fluorescence was recorded during 100ms light flashes at 1kHz. Cytosolic calcium, sodium and pH were calculated as described previously (3-7). Empagliflozin (MedChem Express, Monmouth Junction, NJ, USA) was added from a 5 mmol/L stock solution in 100%DMSO resulting in a maximal of 0.02 % (vol./vol.) DMSO concentration (1 µmol/L Empagliflozin), Cariporide (Aventis Pharma, Frankfurt, Germany) was added from a 5 mmol/L stock solution in HEPES buffer

Measurement of NHE activity in rabbit cardiomyocytes.

NHE activity was measured by recording SNARF-fluorescence (Molecular probes, Eugene, USA;580/640 nm emission; 515 nm excitation) following a NH_4^+ pulse [4]. Before each individual experiment, cells were incubated at 37°C with 10 µmol/L SNARF-AM for 30 min. Myocytes were superfused with HEPES solution ((mmol/L): [Na^+] 156, [K^+] 4.7, Ca^{2+} 1.3, [Mg^{2+}] 2.0, [Cl^-] 150.6, [$HCO3^-$] 4.3, [$HPO4^{2-}$] 1.4, [HEPES] 17, [Glucose] 11, pH 7.3). After 1 min, myocytes were superfused for 10 minutes with the same solution now containing 20 mmol/L NH_4Cl. After 10 minutes the NH_4Cl was removed, which results in an intracellular acidosis. The rate of recovery from this acidosis is a measure of the NHE activity (4). In experiments in which the effect of 1 µmol/L Empagliflozin and 10 µmol/L Cariporide was tested on recovery of acidosis the compounds were added together with NH_4Cl and were present during washout of NH_4Cl. For the glucose free experiment with EMPA, the cells were first pre-incubated in glucose-free medium for 10 min before EMPA was administered.

Mitochondrial calcium.

A ratiometric mitochondrially-targeted FRET-based calcium indicator (4mtD3cpv, MitoCam) was expressed using adenoviral transfection, and the free mitochondrial calcium concentration ($[Ca^{2+}]_m$) was measured using the fluorescence ratio YFP/CFP in 36-48 h cultured adult rat cardiomyocytes (8).

Measurements were performed using an inverted fluorescence microscope at 63x magnification equipped with a photometry setup (IonOptix, Milton, MA, USA). Sarcomere length was determined via spatial fast Fourier transformation (IonOptix, Milton, MA, USA). A 75 W Xenon lamp (USHIO, Tokyo, Japan), set to 65 W, and filtered at 436 nm (bandwidth 20 nm) was used for illumination. A rectangular mask was used to select light emitted from the cardiomyocyte. Emitted light was passed through a long-pass dichroic mirror (T455lp, Chroma Technology, Brattleboro, VT, USA) and fluorescence at 480 nm (CFP) and 540 nm (YFP) was detected using a dichroic mirror and two bandpass filters (ET480/30x and ET540/40m) (Chroma Technology, Brattleboro, VT, USA) and detected with two identical photomultiplier tubes (PMT; H7360-02MOD; Hamamatsu Photonics, Hamamatsu, Japan). Neutral density filters were placed into the light path to avoid saturation. The YFP/CFP ratio calculated after background subtraction is used as a measure of the free intra-mitochondrial Ca^{2+}-concentration (($[Ca^{2+}]_m$).

Measurements were performed at 37 °C in MatTek dishes filled with normal solution containing (in mmol/L): NaCl (134), KCl (5), NaH_2PO_4 (1.2), $MgSO_4$ (1.2), glucose (10), HEPES (10) and $CaCl_2$ (1.8), pH 7.45. $[Ca^{2+}]_m$ at baseline was recorded two minutes after the cessation of 10 electrically-stimulated contractions at a repetition frequency of 0.1 Hz and 1, 5, 10 and 15 min after addition of 1 µmol/L EMPA or vehicle (0.02% (vol./vol.) DMSO). After 15 minutes, cardiomyocytes were stimulated again for 100 s at 0.1 Hz.

All data is available upon request from the authors

1. **Fowler ED, Benoist D, Drinkhill MJ, Stones R, Helmes M, Wüst RCI, Stienen GJM, Steele DS, White E**. Decreased creatine kinase is linked to diastolic dysfunction in rats with right heart failure induced by pulmonary artery hypertension. *J Mol Cell Cardiol* 86: 1-8, 2015.
2. **ter Welle HF, Baartscheer A, Fiolet JWT, Schumacher CA**. The cytoplasmic free energy of ATP hydrolysis in isolated rod-shaped rat myocytes. *J Mol Cell Cardiol* 20: 435-441, 1988.

3. **Baartscheer A. Schumacher CA. Fiolet JWT**. Small changes of cytosolic sodium in rat ventricular myocytes measured with SBFI in emission ratio mode. *J Mol Cell Cardiol* 29: 3375-83, 1997.

4. **Borren v MMGJ, Baartscheer A, Wilders R, Ravesloot JH**. NHE-1 and NBC during pseudo-ischemia/reperfusion in rabbit ventricular myocytes. *J Mol Cell Cardiol* 36: 567-577, 2004.

5. **Baartscheer A, Hardziyenka M, Schumacher CA, Belterman CN, van Borren MM, Verkerk AO, Coronel R, Fiolet.** Chronic inhibition of the Na+/H+ - exchanger causes regression of hypertrophy, heart failure, and ionic and electrophysiological remodeling. *Br J Pharmacol* 154: 1266-75, 2008

6. **Baartscheer A, Schumacher CA, Borren MM, Belterman CN, Coronel R, Fiolet JW**. Increased Na^+/H^+-exchange activity is the cause of increased $[Na^+]$I and underlies disturbed calcium handling in the rabbit pressure and volume overload heart failure model. *Cardiovasc. Res*. 57: 1015-24, 2003.

7. **Baartscheer A, Schumacher CA, van Borren MM, Belterman CN, Coronel R, Opthof T, Fiolet JW.** Chronic inhibition of Na^+/H^+-exchanger attenuates cardiac hypertrophy and prevents cellular remodeling in heart failure. *Cardiovasc Res* 65: 83-92, 2005.

8. **Kaestner L, Scholz A, Tian Q, Ruppenthal S, Tabellion W, Wiesen K, Katus HA, Muller OJ, Kotlikoff MI, and Lipp P.** Genetically encoded Ca^{2+} indicators in cardiac myocytes. *Circ Res* 114: 1623-1639, 2014.
